# Gestational weight gain counselling practices among different antenatal health care providers: a qualitative grounded theory study

**DOI:** 10.1186/s12884-020-2791-8

**Published:** 2020-02-12

**Authors:** Beth Murray-Davis, Howard Berger, Nir Melamed, Karizma Mawjee, Maisah Syed, Jon Barrett, Joel G. Ray, Michael Geary, Sarah D. McDonald, for DOH-NET (Diabetes, Obesity and Hypertension in Pregnancy Research Network) and SOON (Southern Ontario Obstetrical Network) Investigators™

**Affiliations:** 1 Department of Obstetrics and Gynecology, Midwifery Education Program, McMaster Midwifery Research Centre, Hamilton, Ontario Canada; 2 Division of Maternal-Fetal Medicine, Department of Obstetrics and Gynecology, St. Michael’s Hospital, University of Toronto, Toronto, Ontario Canada; 3 Division of Maternal-Fetal Medicine, Department of Obstetrics and Gynecology, Sunnybrook Health Sciences Centre, University of Toronto, Toronto, Ontario Canada; 4 Departments of Medicine and Obstetrics and Gynaecology, St. Michael’s Hospital, University of Toronto, Toronto, Ontario Canada; 5 Department of Obstetrics and Gynaecology, Rotunda Hospital, Dublin, Ireland; 6 Departments of Obstetrics and Gynecology, Radiology and Clinical Epidemiology and Biostatistics, McMaster University, Hamilton, Ontario Canada

**Keywords:** Gestational weight gain, Antenatal care, Counseling, Health care provider perspectives

## Abstract

**Background:**

Inappropriate gestational weight gain in pregnancy may negatively impact health outcomes for mothers and babies. While optimal gestational weight gain is often not acheived, effective counselling by antenatal health care providers is recommended. It is not known if gestational weight gain counselling practices differ by type of antenatal health care provider, namely, family physicians, midwives and obstetricians, and what barriers impede the delivery of such counselling. The objective of this study was to understand the counselling of family physicians, midwives and obstetricians in Ontario and what factors act as barriers and enablers to the provision of counselling about GWG.

**Methods:**

Semi-structured interviews were conducted with seven family physicians, six midwives and five obstetricians in Ontario, Canada, where pregnancy care is universally covered. Convenience and purposive sampling techniques were employed. A grounded theory approach was used for data analysis. Codes, categories and themes were generated using NVIVO software.

**Results:**

Providers reported that they offered gestational weight gain counselling to all patients early in pregnancy. Counselling topics included gestational weight gain targets, nutrition & exercise, gestational diabetes prevention, while dispelling misconceptions about gestational weight gain. Most do not routinely address the adverse outcomes linked to gestational weight gain, or daily caloric intake goals for pregnancy. The health care providers all faced similar barriers to counselling including patient attitudes, social and cultural issues, and accessibility of resources. Patient enthusiasm and access to a dietician motivated health care providers to provide more in-depth gestational weight gain counselling.

**Conclusion:**

Reported gestational weight gain counselling practices were similar between midwives, obstetricians and family physicians. Antenatal knowledge translation tools for patients and health care providers are needed, and would seem to be suitable for use across all three types of health care provider specialties.

## Introduction

Inappropriate gestational weight gain (GWG) is associated with some adverse maternal, fetal and neonatal outcomes. Excess GWG has been associated with gestational diabetes mellitus [1], hypertensive disorders of pregnancy and neonatal macrosomia, whereas inadequate GWG may amplify the risk of fetal growth restriction, and preterm birth [2]. Nevertheless, recommendations for optimal GWG in the 2009 Institute of Medicine guidelines [3], also adopted by Health Canada, are often not met. Studies of Canadian women have found that only 30–35% achieved the recommended weight gain in pregnancy, and over half exceeded the recommended GWG [4–6].

Inappropriate GWG and the associated adverse outcomes can be reduced with lifestyle modifications [7–9]. Yet, these modifications have not been widely implemented. In order for these recommendations and the GWG targets to be operationalized by pregnant women, there needs to be effective guidance from health care providers (HCP). Previous research has explored the connection between antenatal GWG counselling and achieving optimal GWG. Cogswell et al. (1999) found that women who received appropriate advice about GWG were more likely to gain within the recommended ranges [10]. In a randomized controlled trial, investigators found that a greater proportion of women (42.7%) who received tailored nutritional counselling sessions as part of the treatment group gained within the targeted ranges compared to women in the control arm (13.9%) [11]. While the majority of HCPs report counselling women about GWG, 30–40% of women report not receiving counselling [10, 12, 13], and only about a quarter report being informed about risks associated with inappropriate GWG^14^.

There are patient-mediated barriers that impact the association between counselling and optimal GWG, but there are also complex HCP-mediated factors that influence the effectiveness of counselling. HCPs may lack knowledge or may not see it as a priority when there are numerous other issues that need to be addressed during prenatal care [14–16]. Focus groups conducted with antenatal HCPs have suggested that many believe counselling has a minimal impact on women’s health related behaviours [16, 17]. HCPs also expressed concern about providing GWG counselling without discouraging, offending or stigmatizing patients [18]. Self-identified overweight physicians appeared to have greater difficulty counselling about GWG than those of normal weight [15, 19].

It has been suggested that differing models of care among obstetricians (OB), midwives (MW) and family physicians (FP) may influence GWG counselling [20]. Morris et al. (2017) found that MWs more frequently discussed physical activity and food requirements compared to other providers and noted their focus was on overall wellness rather than weight [12]. Yamamoto et al. (2014) found that women seen by OBs were significantly less likely to receive diet and exercise counselling [13]. This may be due to time restrictions since OB appointments typically last only ten minutes, providing less of an opportunity to discuss GWG, compared to appointments with FPs or MWs, which often last 15 and 30–45 min respectively [20]. FPs have also reported that their ability to counsel was impacted by insufficient knowledge and training about nutrition and weight management issues [14].

We were interested in exploring these issues further, including the similarities and differences in counselling of antenatal HCPs such as MWs, OBs, and FPs and the impact the different HCPs’ counselling may have on GWG^4^. The first step undertaken by our research team, reported elsewhere, examined retrospective cohort data from the Better Outcomes Registry and Network (BORN) in Ontario [4]. We found that rates of GWG below, within and above the Institute of Medicine (IOM) recommendations did not differ across HCP groups [4]. Also, there were no differences among the HCPs for the rates of secondary outcomes including large for gestational age or small for gestational age neonates, preterm birth or cesarean section [4]. Next, our research team commenced a qualitative study, reported here, to understand the counselling among antenatal HCPs in Ontario and what factors act as barriers and enablers to the provision of counselling about GWG.

## Methods

We conducted a qualitative, grounded theory study, ethical approval was obtained from the Hamilton Integrated Research Ethics Board.

MWs, FPs and OBs currently providing antenatal care in Ontario, Canada were eligible to participate. Convenience sampling was used for recruitment through the Southern Ontario Obstetrical Network (SOON) (http://www.obgyn.utoronto.ca/gta-obs-network), a group of teaching and community hospitals throughout the Greater Toronto Area and city of Hamilton. Emails were also sent to the heads of service for each discipline at each of the SOON hospitals for distribution. Following this, purposive and snowball sampling were used to increase the variability of participant characteristics, to ensure adequate representation from each profession and to capture a range of experiences from provider groups. Based on previous studies of this nature, a minimum target of five participants from each profession was set, but recruitment continued until saturation was reached, whereby no new information or perspectives were coming forward [21–26].

HCPs completed an initial survey of basic demographic information through an online survey, and following consent, participated in semi-structured interviews conducted over the phone, or in person by a trained research assistant. The research assistant was not a health care provider and has never been pregnant so they did not enter the interviews with any preconceived notions about the counselling process. They were digitally recorded and lasted up to 30 min. The semi-structured interview guide (Additional file 1) was developed by the research team for the purposes of this study.

In keeping with grounded theory, data analysis began at the same time as data collection to make use of the iterative process of constant comparison [21–26]. This constant comparison ensured that the interview questions evolved during data collection to refine the emerging theory.

Interviews were transcribed verbatim and entered into NVivo software. Data analysis began with open coding where words or phrases were used to summarize the essence of the sentence or statement. Open coding of three transcripts was completed by two to ensure consistency in coding. Following open coding of all transcripts, codes were linked to form categories and were then clustered together to form themes. These themes were brought together to develop the emerging theory. Data from all participants was initially analyzed together and further analysis was completed by separating out each professional group to identify similarities and differences.

The research team are from a variety of disciplines, bringing unique perspectives from obstetrics, maternal fetal medicine, and midwifery. The team entered the study holding the belief that gestational weight gain is an important topic and that inappropriate weight gain can result in adverse outcomes for mother and offspring. The researchers who conducted and analyzed the interviews were non-clinicians who had no prior held beliefs about the topic area.

## Results

A total of 18 antenatal HCPs (6 MWs, 7 FPs and 5 OBs) were interviewed. Participant characteristics are outlined in Table 1. Most participants were between 35 and 55 years of age. Years in practice ranged broadly with most having practiced between 1 and 15 years. Sixty percent of participants reported a BMI within the normal range, while the remainder fell into the overweight and obese categories.

**Table 1:** Participant Demographics

Characteristic	Midwife n (%)	Family Physician n(%)	Obstetrician n (%)	Total n (%)
**# of Participants**	6	7	5	18
**Age**				
25–34	1 (16.67)	2 (28.57)	0 (0.00)	3 (16.67)
35–44	4 (66.67)	2 (28.57)	4 (80.00)	10 (55.56)
45–54	0 (0)	1 (14.29)	1 (20.00)	2 (11.11)
55–64	1 (16.67)	2 (28.57)	0 (0.00)	3 (16.67)
**Gender**				
F	6 (100)	85.71	5 (100.00)	17 (94.45)
M	0 (0)	1 (14.29)	0 (0.00)	1 (5.56)
**Highest Level of Education**				
Bachelors	3 (50)	0 (0.00)	0 (0.00)	3 (16.67)
Graduate/Professional	3 (50)	6 (100.00)	5 (100.00)	14 (77.78)
Doctorate	0 (0)	1 (14.29)	0 (0.00)	1 (5.56)
**Years in Practise**				
1 to 5	3 (50)	2 (28.57)	1 (20.00)	6 (33.33)
6 to 10	2 (33.33)	1 (14.29)	1 (20.00)	4 (22.22)
11 to 15	0 (0)	2 (28.57)	3 (60.00)	5 (5.56)
16 to 20	0 (0)	0 (0.00)	0 (0.00)	0 (0.00)
21 to 25	1 (16.67)	0 (0.00)	0 (0.00)	1 (5.56)
26 +	0 (0)	2 (28.57)	0 (0.00)	2 (11.11)
**BMI Categories**				
Underweight < 18.5	0 (0)	0 (0.00)	0 (0.00)	0 (0.00)
Normal 18.5–24.9	1 (16.67)	7 (100.00)	3 (60.00)	11 (61.11)
Overweight 25–29.9	2 (33.33)	0 (0.00)	1 (20.00)	3 (16.67)
Obese 30 ≤	2 (33.33)	0 (0.00)	0 (0.00)	2 (11.11)
No response	1 (16.67)	0 (0.00)	1 (20.00)	2 (11.11)

*Bolded text is used to indicate titles

Our two central themes generated from the findings include:

1. Health care provider counselling practices. This includes addressing topic areas such as gestational weight gain targets, adverse outcomes, GDM, nutrition and exercise counselling and dispelling misconceptions.
2. Barriers and facilitators impacting their ability to provide this counselling. This includes patient and care provider-mediated factors such as sensitivity of the topic area, cultural and financial issues, time, knowledge, availability of resources and perceived impact of counselling.

**Fig. 1:**
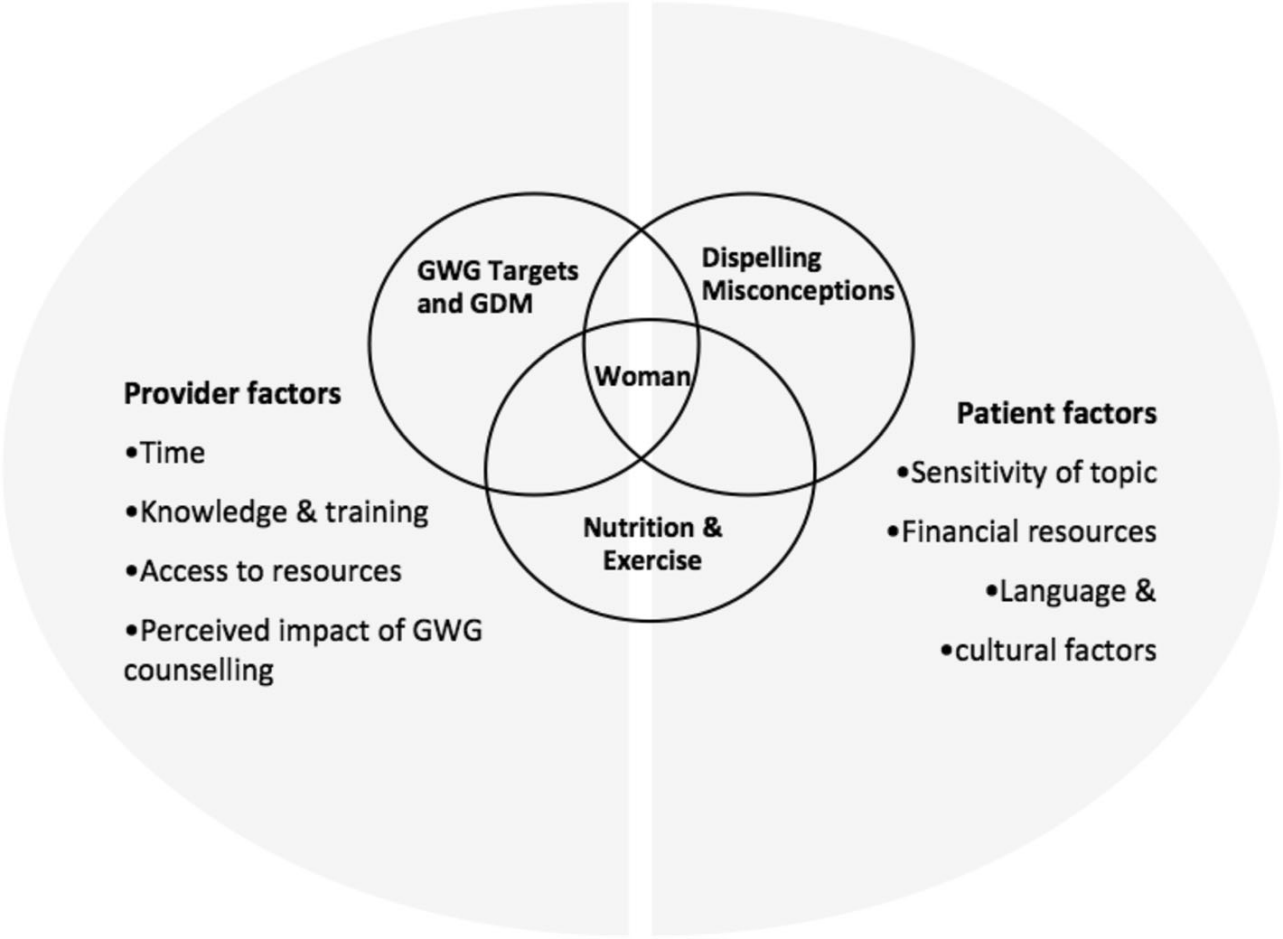
Visual summary of counselling practices and influencing factors. GDM = Gestational Diabetes Mellitus. GWG = Gestational Weight Gain

These are discussed in greater detail below using illustrative quotations with participants identified by a number and initials to denote their profession. Our findings have been summarized visually in Fig. 1, with the topic areas addressed during counselling (theme 1) highlighted in the circles and the barriers and facilitators (theme 2) summarized on in the two half circles. We have placed the woman at the centre of this process.

### Health care providers’ counselling practices

The participants provided an overview of their current counselling practices related to GWG. This included when counselling occurred in the pregnancy, with whom they discussed this topic, and what content areas were addressed. All participants reported that the conversations related to GWG were initiated by them, not by patients. Most reported counselling all of their clients on GWG, however a few suggested they did not address GWG with certain clients, such as those who were late to care. This counselling generally occurred early in pregnancy. Participants described that additional counselling later in pregnancy was not routine and only occurred in certain cases such as when there was excess or inadequate GWG, or if the client requested information. Table 2 contains a summary of the topics included in this counselling.

**Table 2:** Counselling topics addressed by midwives, obstetricians and family physicians regarding GWG

Counselling Topics	Counselling Specific to HCP	Quotes
Gestational Weight Gain Targets ➢ Counselled underweight and overweight women on specific GWG targets. ➢ Provided detailed counselling to women with inadequate or excess GWG. ➢ Counselled those with elevated BMI on aiming for little to no weight gain	➢ MW explained how GWG is distributed over the body during pregnancy. ➢ FPs discussed the amount of GWG to expect on a weekly basis per trimester.	*“I don’t concentrate much on the women who are normal to start off with. But for underweight and overweight, especially my obese population, which we do have a lot, I definitely talk about what the ideal weight gain is and I go with the Institute of Medicine guidelines actually.” – O4*
Nutritional Counselling ➢ Counselled clients to maintain a balanced diet. ➢ Did not provide counselling on caloric requirements. ➢ Gave general counselling on serving sizes.	➢ MWs asked for dietary intake record for 3 days which they then used to provide advice on adjusting diet to meet target GWG goals. ➢ OBs and FPs recommended patients be thoughtful about what they ate, required nutrients for pregnancy and nutritional safety.	*“You do need increases in specific nutrients, so you want to be careful about watching and making sure you’re getting enough protein, you’re getting enough iron, you’re getting enough, so you’re taking your vitamins and you’re being thoughtful about what you’re eating.” – O2*
Exercise counselling ➢ Discussed the importance of regular exercise, dispelled the notion that pregnancy is a time to slow down their physical activity.	➢ OBs and FPs recommended continuing exercise done prior to pregnancy but avoiding starting new exercise activities. ➢ OBs and FPs advised which activities were safe during pregnancy. Most reported not providing specific strategies for getting physical activity. ➢ MWs reported providing specific strategies for staying active. Then would check in at a later visit to review exercise habits.	***“****My basic line for that is to not to start doing anything rigorous, but to continue doing what you’re doing.” – FP6* *“I’ll try to strategize some simple things, let’s say, you know, just small things like if they sit at a desk job all day to suggest like, over your lunch hour get some fresh air and just take a walk around.” – M3*
Adverse maternal and neonatal outcomes ➢ Counselled only those women considered high risk regarding adverse maternal and neonatal outcomes associated with inappropriate GWG. ➢ Counselled about risks only if the patient had inappropriate GWG.	➢ MWs counselled those with excess GWG on increased risk for large babies. ➢ OBs & FPs counseled on the inter-related nature of obesity, gestational diabetes, gestational hypertension, macrosomia and mode of delivery. ➢ OBs counselled those with inadequate weight gain on risk of pre-term birth.	*“I don’t think for the average-risk woman that I talk a lot about pre-term births or underweight babies or macrosomia… I mean we’re generally talking about how all of those issues for women in the higher weight categories are inter-related, right; their risk of high blood pressure, their risk of diabetes that impact on size of baby, impact on mode of delivery, they’re all connected.” – O2*
Gestational diabetes (GDM) ➢ Discussed GDM, generally in response to excess GWG. ➢ Recommeded GDM screening earlier if concerned it was contributing to excess GWG.	➢ MWs discussed strategies for maintaining blood sugar levels. ➢ OBs & FPs referred women with GDM or a high risk of developing it to a dietician.	*“I talk about them not wanting to have their blood sugars go sky high and then drop down. So, I talk about, you know, eating five smaller meals in a day.” – M2* *“Somebody who has GDM, I may explicitly get them to do some more detailed counselling.” – FP1*

MWs, OBs and FPs noted that a key element of their counselling included dispelling misconceptions by cautioning clients about overeating or “eating for two”, and encouraging thoughtfulness about what they ate due to its impact on their baby and their own long-term health and weight. These misconceptions were summarized by one participant who said, *“The grandmother or the parents of the person who is pregnant will say, mom you need to eat more because you’re eating for two. And I say to them, well, the thing you’re eating for is really about the size of your thumb. You don’t need to feed it that much.” – O1.*

When patients presented with inappropriate GWG, participants reported investigating for contributing factors such as extreme nausea or GDM and tailoring the counselling to address these issues. In response to inadequate GWG, providers reported that they offered additional resources, referred patients to dieticians, or prescribed medications to help manage nausea. MWs also reported sharing specific strategies to gain weight:

*“I had a woman who was 100 pounds and she’s not gaining any weight in her pregnancy, and so I talked to her about ways she could add healthy fats to her diet…add more olive oil onto your salads or sprinkle nuts onto your salad*.” *– M8.*

In response to excess GWG, practitioners stated that they reviewed their patients’ dietary and exercise habits to identify areas for improvement and provide further advice. MWs reported that they often responded with more detailed counselling and recommended activities such as keeping a food log. All providers reported that if they suspected GDM was a contributing factor, they recommended GDM testing at an earlier gestational age.

All the HCPs stated using guidelines for GWG targets based on pre-pregnancy BMI. FPs described using websites to share information on eating well and sharing resources on a routine basis. Most MWs and OBs described sharing resources and handouts with clients when inappropriate GWG occurred or if clients asked for additional information.

Referrals for dietary counselling were made when clients were underweight or overweight at the onset of pregnancy, experienced inadequate or excess GWG, suffered from significant nausea, had GDM, had a history of disordered eating, had issues related to food security, or wanted further information on diet counselling.

### Barriers and facilitators for GWG counselling experienced by health care providers

Our thematic analysis highlighted the factors that impacted the HCPs’ ability to address this topic with patients. These included time restrictions, client attitudes, the sensitive nature of the topic, social and cultural concerns, knowledge, and accessibility of resources.

a) “You’re always kind of under the time crunch”.

OBs and FPs reported time limitations as a barrier. The need to address a multitude of routine topics during pregnancy, plus a concern that more women in care present with complicated medical and social issues were seen as factors that restricted the time available. Many OBs and FPs stated that these issues took up the bulk of appointment time and as a result GWG counselling was often omitted:

“*More and more, we have older women who have more complicated medical histories going into pregnancy; and so there’s so much to get through in quite a short visit that it can feel like one of the less important points to cover. And so it can, in those complicated discussions, it can be one of the things that gets dropped first.” – O2*

In contrast, MWs, who have longer appointments, reported sufficient time for detailed counselling and addressing client concerns. This was stated by one MW:

*“I have more time to discuss things with clients. My appointments tend to be 30 to 45 minutes long, whereas a client may only get 5 to 10 minutes with their MD. So, I think for some people that still means that I’m going to say like, this is how much you should gain and that’s the end of the story…But I also have the opportunity to expand if a client is concerned, or if she has, you know, an indication for a greater discussion on it.” – M6*

According to participants infrequent visits in early pregnancy contributed to time barriers for GWG counselling. Some providers, especially OBs, stated that they may only start seeing patients mid-way through their pregnancy, at which time it may be too late to address this issue effectively.

b) “I think much of what we say is not that useful”.

HCPs reported that whether they felt their GWG counselling was impactful, influenced if they were motivated to provide detailed counselling. For example, participants noted that sometimes they perceived their clients to be upset or uninterested in response to this counselling. As described by one OB, “*I think it’s uncomfortable a lot of the time to have a discussion. Some patients are not receptive to it and they get upset with you.” - O1.* Providers noted that particularly when counselling overweight or obese women, they felt them become defensive and that when women have had previous struggles with weight, they seemed less motivated and brush off the topic of GWG. In these instances, providers felt their counselling would be of little impact and felt discouraged to counsel.

Adding to this was the belief that dietary and physical activity choices are habits that have been shaped over many years. Participants felt their counselling would have minimal effect on changing these long term habits. One MW noted this by saying *“I think it’s much deeper rooted psychologically than simply providing some recommended guidelines”. – M8.*

Providers also felt that although their counselling was heard, it was not always taken seriously. In these instances, they thought their counselling was not useful because women already had strong, socially ingrained views about pregnancy. This was described by one OB who stated, “*They don’t want to hear that they can’t eat as much as they want to in the pregnancy”. – O1.*

Many participants felt that the limited GWG counselling they are able to provide would be unlikely to overcome these views and have an impact.

Conversely, providers reported that when they sensed a client was eager to hear their counselling, they felt encouraged to continue. This gave them a sense that their GWG counselling was impactful and thus was important. HCPs also noted that dispelling misconceptions about pregnancy, seemed to have an impact. This was noted by a MW who said:

*“I think there’s still some sort of cultural ideas about when women are pregnant…I have this really normal excuse to need to eat more or to treat myself to ice cream. And so I think sometimes in those cases actually that can be… where you actually talk about, like, actually this is maybe a time in life where you might want to be more careful” – M3*

c) “You have to be very thoughtful about how to frame it”.

HCPs described that sensitivity and stigma related to the topic of weight gain was a significant barrier to offering appropriate counselling. Participants reported feeling uncomfortable bringing up their patient’s weight with them, fearing that it would not be received as a discussion about a medical concern and instead as a personal judgement or attack on their life decisions. Participants described that a certain amount of rapport needs to be built to overcome this challenge. However, OBs and FPS reported that this can be difficult as a result of their limited time during appointments. In contrast, MWs stated that longer appointments allowed opportunity to build a relationship with their clients.

Participants also discussed how their own weight influenced their confidence to counsel on GWG. For example, one participant described:

*“As like a chubby person myself…I sometimes feel when I am chatting to other women who are overweight or obese, I sometimes feel like I have some sense of I can, you know, empathize with them, and say like, ‘I know these are hard changes to make and many of us have a hard time doing that.’…And at the same time, sometimes I feel a bit hypocritical.”* – M3

MWs discussed not wanting to contribute to the existing pressures women faced regarding their weight, and described being sensitive to this as part of their approach to care. They mentioned that by discussing GWG excessively, they feared they may create anxiety related to weight and aggravate existing body image issues. For example, one MW noted, *“The counselling of the midwives in my practice is that we are very well aware of how weight is a huge issue for women. So we’re very focused on not making people feel guilty and encouraging and praising them when they do well”. – M9.*

To manage the sensitivity around this topic, MWs reported giving their clients the option of self-weighing in order to shift women’s focus from the amount of weight gained to having a balanced diet.

d) “Some clients just really can’t afford a lot of nutritious foods”.

OBs and FPs reported that financial limitations were sometimes a barrier for accessing dieticians, because connecting with dieticians externally whose services were covered by OHIP was challenging. And, when dieticians were not covered by insurance or OHIP, many women were unable to pay out of their pocket. As a result, participants reported that they become selective with who they refer for this service: *“At my other clinic, the dietitian isn’t covered, so for patients who have benefits, then I bring it up as an option, but for patients who don’t, I don’t always.” – FP5.*

OBs and FPs described that having access to dieticians and GDM clinics within their practices whom they could make referrals to was a support and supplement for their counselling, especially when addressing complex issues.

Differences in cultural background and language were also sometimes a barrier. Even with the support of an interpreter, providers described that they couldn’t be certain what was being passed to the client and how that was being received. One FP suggested that this could potentially be overcome with the use of handouts containing pictures of foods from many cultures.

Participants collectively noted the need for an increase in the number of affordable classes and groups for women to attend, focused on being healthy and staying active, and classes led by dieticians.

e) “I was not well trained to have these conversations”.

Most participants reported receiving inadequate knowledge and training related to GWG during their formal education. Often during their training, participants observed minimal to no counselling on GWG. Therefore, they felt hesitant due to feeling ill-equipped. One FP articulated this by saying, **“***Well, I’m a pretty old doc and I don’t think anybody ever taught me anything about that, to be honest… I don’t think I’ve had special training on nutritional necessities in pregnancy.” – FP3.*

Providers mentioned that areas in which they specifically lacked knowledge included, caloric requirements for pregnancy, and appropriate diet and exercise strategies. Some HCPs acknowledged that information on the adverse outcomes of inappropriate GWG would be a strong motivator for clients to manage their GWG but they did not always feel adequately prepared to share this information. This was described by one MW who stated *“I feel like there are probably colleagues of mine who are better educated than I am on the effects of weight gain in pregnancy; so, I feel like I probably could use some more education on it.” - M2.*

Many also reported that they had not taken steps to improve their knowledge on GWG and noted that although current evidence had evolved, they had not updated themselves with this new information. One participant stated *“I have been practicing more than 20 years, so I do feel I’m not as up to date on current information as I could be.” – M9*. To overcome this barrier, participants echoed a need for more standardized guidelines.

A few HCPs mentioned that they lean on the knowledge they’ve gained from their own interests and experiences in personal health, pregnancy and GWG to provide appropriate counselling. Moreover, when GWG is a priority in their profession or practice, providers stated they were motivated to provide counselling and increase their knowledge in this area. This was mentioned particularly by OBs working in fertility practices where obesity may impact fertility.

Nf) “I don’t necessarily have the stuff handy”.

Participants reported that more accessible information and resources wre needed for counselling. Since resources for providers were limited, participants stated that they had to develop these resources themselves, access online resources during appointments, or rely on their memory. One FP stated, *“If we don’t have the information at our fingertips, which sometimes is the case….that’s a time issue again, just needing to sort of say, let me just sort of check this website”. – FP1* Providers suggested developing resources such as flashcards or leaflets with pictograms.

## Discussion

We hypothesized that the counselling provided to pregnant women in Ontario would be variable among different types of antenatal HCPs. However, our results indicated that in fact, MWs, OBs and FPs had similar counselling practices. The majority of providers counseled most patients early in pregnancy. Additionally, the topics covered were largely consistent between the different HCPs with a few variations. This included information on GWG targets, general nutrition and exercise counselling, information for high risk women on the adverse maternal and neonatal outcomes linked to inappropriate GWG, gestational diabetes, and dispelling misconceptions about pregnancy.

Despite this comprehensive list of topics related to GWG, it would appear that there are two critical gaps in the counselling provided to patients. First, despite the short-term, long-term and intergenerational implications, a discussion of why appropriate weight gain is important, including an overview of the adverse outcomes for mothers and babies was not routinely provided for patients, unless they already had a pre-pregnant BMI above or below recommendations, or their GWG was inappropriate. These findings are in line with other studies in the literature showing similar gaps in the GWG counselling [12, 20].

Second, the results of our study indicated that there was a lack of information on specific strategies for eating healthy and exercising in pregnancy as part of GWG counselling. This is consistent with findings by Morris et al. (2017) and Yamamoto et al. (2014) [12, 13]. The importance of providing specific nutrition and exercise strategies during counselling was highlighted in a systematic review by Vanstone et al. (2016) exploring women’s perceptions of GWG^18^. They found that women lacked understanding of how to operationalize general counselling advice from their HCP such as “eat healthy” [18]. Women reported that they required practical tips such as quick, healthy and inexpensive meal ideas, ways to decrease impulse food decisions, and how to individualize nutritional advice based on allergies, food preferences and culturally-specific meals [18]. Similarly, women reported that without receiving counselling on specific exercise strategies, they did not know suitable exercises, nor the intensity or duration of exercises that were appropriate for pregnancy [18].

Findings from our study demonstrated that MWs, OBs and FPs in Ontario faced similar barriers when counselling on GWG. These included patient related factors such as the perceived sensitivity of the topic, financial and cultural barriers. Provider related factors included knowledge and accessibility of resources as well as the perceived impact of GWG counselling. The care providers demonstrated limited awareness of, or use of available resources such as those created by Health Canada. OBs and FPs experienced the additional barrier of time restrictions due to shorter appointment length. These findings are in keeping with existing literature on the topic. For example, Stotland et al. (2010) identified insufficient training and concern about the sensitivity of GWG as barriers for GWG counselling [14], while Whitaker (2016) identified that lack of time, cultural differences and lack of patient interest prevented adequate counselling [19]. Additionally, Chang et al. (2013) reported that providers believed their counselling had low impact on patients’ weight gain and that GWG is more influenced by factors such as family, habits and culture [16]. More research is needed to examine the impact of cultural factors that may impact gestational weight gain and healthy habits in pregnancy.

The identification of the common barriers experienced by the HCPs indicates that strategies to improve providers’ ability to counsel on GWG are needed and understanding these perspectives is a necessary first step before developing interventions to address the barriers. Although previous studies have indicated that short appointment lengths may be an important factor resulting in inadequate GWG counselling and inappropriate GWG among pregnant women [19, 20], results from this study along with our retrospective study [4] indicate otherwise since similar rates of inadequate, appropriate and excess GWG were found across MW, FP and OB patient populations [4]. This suggests that longer appointment lengths may not be the answer for improving GWG counselling practices among HCPs. To our knowledge there has been one intervention study to date that examined tools for HCPs for GWG counselling. Further research is needed in this area, since effective resources such as the 5A’s Approach (Ask, Assess, Advise, Agree, Assist) exist, but don’t appear to be influencing daily practice [27, 28]. There is growing evidence outside of obstetrics that behaviour change counselling, combined with point of care tools about obesity, may result in patient behavior change and improved outcomes, while at the same time improving initiation of the discussion by clinicians [29]. Our findings indicate that care providers are simply providing information and advice and are not making use of counselling “techniques” such as cognitive behavior change therapy, or motivational interviewing which may be more likely to result in behavior change.

Our study highlighted discordance with IOM guidelines which recommend HCPs advise patients on GWG targets, track and discuss GWG throughout pregnancy, and offer tailored counselling on nutrition and physical activity [3]. It appears from our interviews that discussion of GWG targets has become part of routine practice, but on-going and individualized counselling is not consistently offered. Further, up to date information on nutritional and physical activity requirements, and the adverse outcomes associated with inappropriate GWG would be important to assist providers. Future research should consider the development and evaluation of interventions and knowledge translation strategies and tools for HCPs.

Strengths of our study included the multidisciplinary nature of our research team; permitting us to consider GWG counselling from the different provider perspectives. A limitation of the study is that views of antenatal care providers in Ontario may not represent those in other jurisdictions. Also, although we employed recruitment techniques to ensure a range of experiences were represented in each of the HCP groups, we recognize that practitioners who are more interested in GWG counselling to other practitioners were likely to have chosen to participate in our study. Further, we did not collect demographic information about the care providers ethnic and cultural background. This would be useful for future research given that cultural backgrounds and beliefs were identified as factors informing healthy behaviours.

## Conclusion

Findings from our study indicated that reported GWG counselling practices were similar between MWs, OBs and FPs providing antenatal care in Ontario. Additionally, barriers for counselling were consistent across the providers, although MWs, unlike the other groups, did not feel time was a barrier. Adequate knowledge about excess GWG’s effects is an on-going challenge for HCPs and highlights the need for the development and evaluation of knowledge translation tools to effectively address this topic during antenatal care.

## Supplementary information

**Additional file 1.** Interview Guide

## Data Availability

The datasets used and analysed during the current study are available from the corresponding author on reasonable request.

## Abbreviations

BORN: Better Outcomes Registry and Network
FP: Family physicians
GDM: Gestational diabetes
GWG: Gestational weight gain
HCP: Health care providers
IOM: Institute of Medicine
MW: Midwives
OB: Obstetricians
SOON: Southern Ontario Obstetrical Network
