# Supplementary material for: Gestational weight gain counselling practices among different antenatal health care providers: a qualitative grounded theory study
Source: BMC Pregnancy Childbirth. 2020 Feb 12;20:102. doi: 10.1186/s12884-020-2791-8 (PMC7017610; doi:10.1186/s12884-020-2791-8)
Supplement: Supplementary file 1 — Additional file 1. Interview Guide [file 12884_2020_2791_MOESM1_ESM.docx]

**Interview Guide**

1. **Can you begin by describing how you typically counsel your women about gestational weight gain?**

- ***Timing?***

1. When do you initiate this discussion?
2. Are there other times during pregnancy that you discuss this topic?

- ***Who initiates?***

1. Who initiates this discussion – you or the woman? Or someone else in your office?

- ***Content?***

1. What content do you typically cover in this discussion?
2. In what way do you address the following:

-Specific amount of weight gain?

-Specific calorie count?

-General nutrition information?

-Exercise?

- ***With whom do you address this?***

All women, only those who bring it up, only those who you think would benefit?

1. **In what ways do you think the counselling you provide is the same or different than other health care providers in pregnancy?**
2. Is this the same or different than colleagues in the same profession?
3. Is this the same or different than other health care providers such as midwives/family doctors/obstetricians
4. **Are there other resources or sources of information on gestational weight gain that you recommend to your clients/patients?**
5. **What are some of the barriers that might prevent you from counselling women about gestational weight gain?**
6. How does your time availability/management impact your ability to have these discussions?
7. How does your knowledge and training on the topic area impact your ability to have these discussions?
8. **How effective do you think counselling on gestational weight gain is for helping women gain an appropriate amount of weight?**
9. **How can health care providers help women to receive and retain information on gestational weight gain?**
10. **What other strategies would help women to gain within the recommended ranges during pregnancy?**
11. **How do you typically respond when women have abnormal weight gain (either above or below recommended ranges)?**
12. Do you change your counseling approach? If yes, how?
13. Do you change your care plan for assessments or management? If yes, how?
